# Effects of gamma-radiation on microbial, nutritional, and functional properties of Katimon mango peels: A combined biochemical and in silico studies

**DOI:** 10.1016/j.heliyon.2023.e21556

**Published:** 2023-10-31

**Authors:** Tabassum Jabin, Suvro Biswas, Shirmin Islam, Swagotom Sarker, Mirola Afroze, Gobindo Kumar Paul, Mamudul Hasan Razu, Md Monirruzzaman, Mainul Huda, Mashiur Rahman, Nayan Kumer Kundu, Sabiha Kamal, Pranab Karmakar, Md Ariful Islam, Md Abu Saleh, Mala Khan, Shahriar Zaman

**Affiliations:** a Microbiology Laboratory, Department of Genetic Engineering and Biotechnology, University of Rajshahi, Bangladesh; b Bangladesh Reference Institute for Chemical Measurements (BRiCM), Dhaka, Bangladesh

**Keywords:** Gamma radiation, Antimicrobial activities, Mango peels, Biochemical assay, In silico

## Abstract

Gamma radiation has notable impacts on the flesh of mangoes. In this research, Katimon mangoes were subjected to different levels of irradiation (0.5, 1.0, 1.5, and 2.0 kGy) using a^60^Co irradiator. The results showed that irradiation significantly reduced the microbial population in the mango peels, with the 1.5 kGy dose showing the most significant reduction. Irradiation also delayed ripening and extended the shelf life of the mango peels. The total fat, protein, ash, moisture, and sugar content of the mango peels were all affected by irradiation. The total protein content, ash content and moisture content increased after irradiation, while the fat content remained relatively unchanged. The sugar content increased in all samples after storage, but the non-irradiated samples had higher sugar levels than the irradiated ones. The dietary fiber content of the mango peels was not significantly affected by irradiation. The vitamin C content decreased in all samples after storage. The titratable acidity and total soluble solids content of the mango peels increased after storage, but there were no significant differences between the irradiated and non-irradiated samples. Antioxidant activity and cytotoxicity assessment highlighted the antioxidant potential and reduced toxicity of irradiated samples. Additionally, the antimicrobial effectiveness of irradiated mango peels was evaluated. The most substantial inhibitory zones (measuring 16.90 ± 0.35) against *Pseudomonas* sp. were observed at a radiation dose of 1.5 kGy with 150 μg/disc. To identify potential antimicrobial agents, the volatile components of mangoes irradiated with 1.5 kGy were analyzed through GC-MS. Subsequently, these compounds were subjected to in silico studies against a viable protein, TgpA, of *Pseudomonas* sp. (PDB ID: 6G49). Based on molecular dynamic simulations and ADMET properties, (−)-Carvone (−6.2), *p*-Cymene (−6.1), and Acetic acid phenylmethyl ester (−6.1) were identified as promising compounds for controlling *Pseudomonas* sp.

## 1 Introduction

Mango fruit production, trade, and consumption have experienced significant growth both locally and globally due to the fruit's appealing nutritional profile [1]. Mango (*Mangifera indica* L., Anacardiaceae) holds a prominent position among tropical fruits worldwide. The genus *Mangifera* comprises approximately 69 species, primarily found in tropical Asia [2]. Mangoes are processed into various products like puree, canned slices, syrup, nectar, pickles, chutney, and jam [3]. In Bangladesh, the all-season mango varieties such as BARI-11, Katimon have become popular. The adoption of fruit bagging techniques has contributed to the safety and enhanced flavor of mangoes in recent times. The Thai mango variety ‘Katimon’ is available year-round in Rajshahi, extending the previous harvesting window from approximately three and a half months, from May to mid-August. This high-yielding mango variety is well-suited for cultivation throughout Bangladesh and holds promise for profitability [4].

Recent findings have revealed that agro-industrial byproducts contain active compounds suitable for human consumption [5]. Mango peels have garnered attention from the scientific community due to their rich content of valuable compounds, including phytochemicals, polyphenols, carotenoids, enzymes, vitamin E, and vitamin C, which offer functional and antioxidant benefits. Mangoes are also rich in soluble and insoluble dietary fiber and phytochemicals crucial for maintaining good health [6]. With the rise in antibiotic resistance among microorganisms and the persistence of adverse effects associated with synthetic medications, interest in alternative therapies has resurfaced. Plant-based remedies were the primary form of healthcare before the emergence of modern medical systems [7]. Mango fruit peels contain phenolic compounds with antioxidant, antimicrobial, and colorant properties, some of which serve as natural defenses against pathogens and environmental conditions [8]. Several techniques are employed to ensure the microbiological stability and sanitary quality of fresh-cut fruits [9].

Food irradiation stands as an economically viable approach to minimize post-harvest losses, prolong the shelf life of perishable goods, enhance food hygiene, and deactivate foodborne viruses and parasites [10]. Gamma radiation has an impact on the total phenol concentration and may elevate the tannin content of extract compounds [11,12]. The antibacterial properties are associated with phenolic compounds, particularly tannins. These effects stem from tannins' ability to hinder bacterial enzymes and interact with enzyme substrates [11,13].

Volatile components constitute a diverse group of compounds with distinct chemical roles that contribute to the aroma of the fruit [14]. Mango peels have proven to be a potent source of flavor, containing odor-active chemicals in higher concentrations than the edible portions of the fruit. This discovery has led to a renewed interest in utilizing mango peels as a natural source of flavorings, capitalizing on the recognized sensory qualities of mangoes [15]. However, conventional methods for identifying potential bioactive phytochemicals involve time-consuming sample preparation, expensive chemicals, and rigorous procedures [16]. To address these challenges, the in-silico approach has gained traction in biotechnology, pharmaceuticals, and bioinformatics. While further research is needed to enhance the precision and accuracy of predictions, this bioinformatics method holds promise for tasks such as identification, computation of quantitative descriptors, toxicity prediction, and analysis of interactions between phytochemicals and target enzymes [17].

This study aims to evaluate the nutritional attributes of mango peels for potential use in various meals and to investigate the antimicrobial properties of differently gamma radiation-exposed mango peels, both in vitro and in silico conditions.

## 2 Materials and methods

### 2.1 Sample collection

On 21 August 2021, Katimon mangoes were obtained from Ambagan in Chapainawabganj, Bangladesh. These mangoes were fresh, green-yellowish in color, and free from bruises. The selection criteria focused on consistent size, shape, and maturity. The mangoes were transported to the Bangladesh Atomic Energy Commission in Savar, Dhaka. After being washed and air-dried, each mango was individually encased in breathable thin polythene bags. High Density Polyethylene (HDPE) bags with a thickness of 30 μm were used to preserve the mangoes.

### 2.2 Gamma irradiation

The following day, at the Institute of Food and Radiation Biology (IFRB), Bangladesh Atomic Energy Commission in Dhaka, a Co-60 gamma cell was used to administer gamma irradiation treatment. The applied dose rate was 5.81 kGy/h. Various doses of irradiation (0.5, 1.0, 1.5, and 2.0 kGy) were administered to the mangoes. A control group of mangoes without irradiation was also included. Both irradiated and non-irradiated mangoes were placed in a humidity cabinet at 19 ± 2 °C for several days.

### 2.3 Total microbial count and ripening evaluation

Microbial populations were isolated from the mangoes using a washing and dilution plating procedure. A single mango was mixed with a specific amount of sterilized distilled water in a beaker and shaken for 15 min. The resulting mixture was diluted, and 0.1 ml samples from each dilution were spread on nutrient medium plates and incubated at 35 °C for 24 h [18]. After incubation, the colonies on the plates were manually counted and reported as colony-forming units per milliliter (CFU/ml). Multiple copies of both the control and irradiated samples were examined.

The assessment of ripening involved monitoring the color and texture of the mangoes stored in a humidity cabinet at 19 ± 2 °C [19]. A systematic approach was adopted to observe and document the physiological and morphological changes in the fruits before and after ripening, with and without gamma radiation treatment. Organoleptic characteristics like skin color, shriveling, aroma, pulp color, flavor, and taste were evaluated using the hedonic scale method. This ensured a comprehensive analysis of the sensory attributes of the fruits [20].

### 2.4 Sample preparation

During the first and second days of storage, the mango peels were removed using a sterilized knife. For specific analyses, some mango peels were homogenized using a clean grinder. Before being ground into powder for subsequent tests, the peels were air-dried at room temperature for about a month. The required amount of dried peel was precisely weighed for different analyses. Each experiment was conducted three times to ensure reliable results for each parameter.

### 2.5 Assessment of nutritional characteristics

The fat content was determined using the AOAC (Association of Official Analytical Chemists) method, while the protein percentage in Katimon mango peel samples was analyzed using a technique described in the AOAC book with minor adjustment [21]. For ash determination, a sample was heated at 600 °C for 6 h until a consistent weight was reached [22]. The moisture content and the total sugar content of mango peel samples were measured using the AOAC official method. To compute the total dietary fiber in a mango peel sample, modifications were made to the AOAC technique. Vitamin C was determined using the 2,6-dichlorophenolindophenol visual titration method, as per AOAC guidelines. The total titratable acidity (TTA) of a food sample indicates its acid content. Titratable acidity in Katimon mango peels was gauged using the AOAC approach [21].

The total carbohydrate content in mango peel samples was calculated using the following equation [22]:

Totalcarbohydrate(%)=100‐{Moisture(%)+Protein(%)+Fat(%)+Ash(%)}

The gross dietary energy was calculated using the total carbohydrate, protein, and fat values in the samples as indicated by the following equation:

FE={(%TCx4}+(%TFx9)+(%TPx4)

Here, TC- Total carbohydrate.

TF-Total fat.

TP-Total protein.

### 2.6 Extraction of mango peels

Mango peel extracts were prepared using a method outlined by Janarthanam et al. [23] with minor modifications. In 1 L conical flasks, 100 g of peel powder was used for extraction. Each flask was treated with 500–600 ml of 95 % methanol. These flasks were sealed and placed on an orbital shaker at 150 rpm for two days at 37 °C. Afterward, the mixture was filtered using Whatman No. 1 filter paper. The methanolic extracts were evaporated using a rotary evaporator at 50–60 °C and a rotation speed of 160–180 rpm. After about 30 min of drying, concentrated slurry was obtained and stored in small vials for further drying. Solvents were evaporated over a period of 20–30 days, resulting in ready-to-use extracts.

### 2.7 Antimicrobial activity assay

This study utilized *Pseudomonas* sp., *Escherichia coli*, and *Salmonella* sp. bacterial strains. Extract solutions from the previously prepared *M. indica* samples were applied to paper discs in volumes of 25 μl, 50 μl, 100 μl, and 150 μl using a micropipette. Thus, the paper discs contained 25 μg, 50 μg, 100 μg, and 150 μg of peel extract, respectively. Kanamycin (30 μg/disc) was used as a control disc. These discs were placed on LB agar plates seeded with the test bacterium and incubated at 37 °C overnight. The width of inhibitory zones was measured in millimeters using a clear scale to determine antibacterial activity.

### 2.8 Antioxidant activity test

The IC_50_ value for Butylated hydroxytoluene (BHT) DPPH scavenging ability was initially determined [24]. Test tubes labeled with concentrations 1, 2, 3, and a control were prepared. From the stock solution of *Mangifera indica* peel extract, solutions of 10 μl, 20 μl, and 30 μl were added to the respective test tubes. The control tube received no extract solution. Solvent (methanol) was added to achieve a total volume of 990 μl, 980 μl, 970 μl, and 1000 μl in the first, second, third, and control tubes, respectively. DPPH solution was added, and the tubes were incubated in the dark at room temperature for 30 min. The absorbance was measured at 519 nm using a UV spectrophotometer. Similar to the BHT experiment, the IC_50_ value and percentage of DPPH scavenging activity were calculated for the extract.

### 2.9 Cytotoxicity test on brine shrimp

Brine shrimp nauplii were subjected to *M. indica* peel extracts for toxicity testing. Different concentrations (50, 100, and 150 μl) of the previously prepared *M. indica* peel extract stock solution were added sequentially to three test tubes using a micropipette. The control test tube was devoid of the extract solution. Methanol was added in increasing quantities (950, 900, 850, and 1000 μl) to each test tube. Each test tube was then filled with 10 ml of artificial seawater containing 10 nauplii. The tubes were left at room temperature for 24 h for observation.

After a day, the vials were examined, and the viable nauplii count was performed for each vial. The results were documented in a logbook, allowing for the determination of the mortality rate of brine shrimp nauplii at each concentration for every sample [25].

### 2.10 Volatile compound analysis through GC-MS

Due to the predominantly positive outcomes for 1.5 kGy-irradiated mangoes, volatile compounds in both the 1.5 kGy irradiated mangoes and the control samples were identified. Optimal Solid-Phase Microextraction (SPME) experimental settings were established based on previous studies [26]. About 5.0 g of sample and 1.5 g of NaCl were combined in a 15 ml vial with a PTFE-silicon septum seal, agitated at 80 rpm. Extraction temperature was set at 40° Celsius. A conditioned SPME fiber (pre-treated at 270 °C for 30 min) was introduced into the headspace after the sample vial equilibrated at 40 °C for 10 min on a heated platform with agitation. Extraction was carried out for 30 min with continuous heating and agitation.

The fiber was then removed and immediately placed in the GC for desorption and analysis. GC-MS analysis employed an Agilent 7890 gas chromatography system and an Agilent 5977 mass selective detector. HP-5 and DB-WAX columns (both 30 m 0.25 mm i.d., 0.25 mm film thickness) facilitated compound separation. Electron ionization mode (EI) with a 70-eV ionization energy was used. Volatile compounds were identified using authentic standards, retention indices (RI), and the NIST 14.0 library. Compound retention indices (RIs) were determined by injecting a homologous sequence of straight-chain alkanes (C6–C30) into a sample.

### 2.11 Ligand preparation

To prepare the ligands, the SDF format of three-dimensional structures was retrieved from the PubChem database (https://pubchem.ncbi.nlm.nih.gov/) [27] for the volatile compounds of *Mangifera indica* (Katimon 1.5 kGy) identified by GC-MS chromatogram. For the construction and improvement of the ligand structures, Avogadro software's mmff94 force field was used [28].

### 2.12 Protein preparation

The Protein Data Bank (https://www.rcsb.org/) was used to retrieve the structure of the TgpA protein (a key protein for *Pseudomonas* sp. viability; PDB ID: 6G49). As part of the protein structure preparation, heteroatoms and water molecules were removed from the protein using BIOVIA Discovery Studio [29]. As well as, in YASARA software [30], the AMBER14 force field [31] was used to minimize the energy of the protein structure. This energy-minimized protein structure was used for molecular docking and dynamics studies.

### 2.13 Molecular docking study

A virtual screening tool, the PyRx was performed to get the best knowledge about the binding affinity and interaction between the potential ligand and the TgpA protein, and molecular docking was done in association with AutoDock Vina protocol [32]. Every single ligand was converted into PDBQT format making them in an acceptable format for the further step to employ molecular docking. The universal force field (UFF) was used to minimize every ligand-free energy value. In this process, total bonds were allowed to be rotated as the docking configuration was protein-fixed and ligand-flexible. A grid box was formed having the center point set with X: −9.2528, Y: −23.484, and Z: −17.9691, and the dimensions (in Å) were X: 49.3688, Y: 44.3499, and Z: 57.8486. The ligand binding affinity values were expressed in negative kcal/mole units where the lowest binding affinity indicated the best confirmation. Afterward, PyMol [33], UCSF Chimera [34], and BIOVIA Discovery Studio were used to visualize the non-bonding interactions.

### 2.14 ADMET analysis

Consistent online servers including admetSAR [35], SwissADME [36], and pKCSM[37] were used to predict absorption, distribution, metabolism, excretion, and toxicity; in short ADMET properties to examine the pharmacokinetic characteristics of the top three docked molecules. The canonical SMILES (simplified molecular-input line-entry system) of the top three phytochemicals were obtained from the PubChem database and entered into these web servers for drug-likeness properties prediction.

### 2.15 Molecular dynamics simulation

The molecular dynamics simulation (MD) regarding the ligand-protein complexes was conducted by utilizing the YASARA dynamics software programs [30,38], in tandem with the AMBER14 force field [39,40]. Later, the hydrogen bond-based network regarding the preferred docked complexes was subjected to optimization and orientation along with prior initial cleaning. A cubic simulation cellular compartment in conjunction with systematic boundary impediments was created by utilizing the TIP3P solvation model [[41], [42], [43]]. The simulation compartment was subjected to 20 Å per direction elongation exceeding the ligand and protein complex. The physiological parameters regarding the simulation cell incorporate 0.9 % NaCl concentration, 298 K, and pH 7.4. During the simulated annealing approach, the steepest gradient algorithms including 5000 cycles were executed for the preliminary energy minimization [44,45]. The time step regarding the simulation strategy was accommodated to 1.25 fs. The PME or Particle Mesh Ewald system along with an 8.0 Å cutoff radius was utilized for the calculations of the long-range electrostatic interactions [31,[46], [47], [48]]. Later at every 100 ps, the simulation trajectory data was kept. A constant pressure, temperature, and a Berendsen thermostat were maintained during the simulations conducted for 100 ns [49,50]. The analysis concerning the root mean square deviation (RMSD), root mean square fluctuation (RMSF), solvent accessible surface area (SASA), radius of gyration (Rg), and hydrogen bond was conducted by assessing simulation trajectories [[51], [52], [53], [54]].

### 2.16 Statistical analysis

The experimental setup was fully randomized with three repetitions. The experimental data were analyzed using one-way analysis of variance (ANOVA) in SPSS, and significant differences between treatment means were compared using Duncan's Multiple Range Test (DMRT). Graphical figures were created using GraphPad Prism 8.

## 3 Results

### 3.1 Total microbial count

Table 1 shows that after a two-week observation period, the microbial population in all irradiated samples was lower than that in the control. The microbial count in the control sample of Katimon mango was 5.3 × 10^5^ CFU/ml. The microbial burden in the sample was significantly higher. In comparison, the microbial load in the 1.5 kGy irradiated Katimon sample was 2.1 × 10^2^ CFU/ml in the first week and 4.1 × 10^2^ CFU/ml in the second week (Table 1).

**Table 1:** Total microbial count of control and irradiated mangoes (0.5 kGy, 1 kGy, 1.5 kGy, and 2 kGy) stored at 4 °C for two weeks. Different letters used to indicate significant differences between the mean ± SD of replications (n = 3) at a significance level of P < 0.05.

Gamma Irradiation (kGy)	Microbial Load on Nutrient Agar (CFU/mL) Analysis Period (Weeks) Katimon	
	Week 1	Week 2
**Control**	5.3 × 10^5^±0.55^a^	7.3 × 10^7^±0.7^a^
**0.5 kGy**	3.4 × 10^3^±0.6^b^	4.4 × 10^4^±0.6^b^
**1 kGy**	2.8 × 10^2^±0.75^b^	2.7 × 10^3^±0.79^b^
**1.5 kGy**	2.1 × 10^2^±0.47^b^	4.1 × 10^2^±0.91^b^
**2 kGy**	1.9 × 10^2^±0.47^b^	3.6 × 10^2^±0.57^b^

### 3.2 Ripening evaluation

Throughout the initial week, the texture of both the control and treated samples (0.5 kGy, 1 kGy, 1.5 kGy, and 2.0 kGy) remained relatively unchanged (Table 2). The control mangoes began to undergo ripening and softening after the second week, evident from the change in peel color to yellow. By the end of two weeks, the control mangoes had decayed. Among the treated samples, mangoes exposed to a dose of 2.0 kGy were excessively soft, while those irradiated at 1.5 kGy displayed a firmer texture (Table 2).

**Table 2:** Ripening and sensory evaluation of control and irradiated mangoes (Katimon).

Gamma Irradiation (kGy)	Ripening Evaluation (Katimon)			
	Week 1		Week 2	
	Texture	Color	Texture	Color
**Control**	Firm	Green	Overripe	Yellowish
**0.5 kGy**	Firm	Green	Less firm	Green
**1 kGy**	Firm	Green	Firm	Green
**1.5 kGy**	Firm	Green	firm	Green
**2 kGy**	Firm	Green	Less Firm	Yellowish

### 3.3 Total fat content

**Fig. 1:**
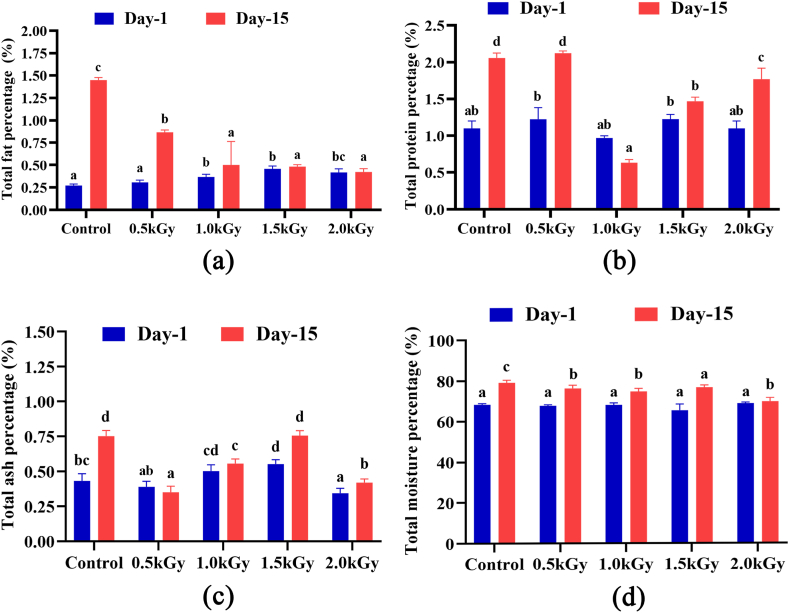
Total fat, protein, ash and moisture percentage of the peel extract on day 1 and day 15. Different letters indicate significant differences between the mean ± SD of treatments (n = 3) at a P < 0.05 significance level.

The total fat content of mango peel samples was measured on day one and after a 15-day storage period. The control Katimon peel exhibited the lowest fat content of 0.27 %, whereas the 1.5 kGy irradiated sample demonstrated the highest fat content at 0.45 % on day one. After 15 days, the fat content in the control increased significantly (1.44 %) due to over-ripening. However, the radiation-exposed samples exhibited no significant changes (Fig. 1a).

### 3.4 Total protein content

Among the various doses, the Katimon mango irradiated with 1.0 kGy had the lowest protein content at 0.97 %. After 15 days of storage, noticeable protein level fluctuations were observed in the control and 0.5 kGy irradiated samples, while the other samples showed minimal alterations. Overall, the protein content increased in all samples except in the sample irradiated at 1.0 kGy (Fig. 1b).

### 3.5 Total ash content

On day one, the ash content in Katimon mango irradiated with a 2 kGy dose was lower (0.34 %). Following a 15-day storage period, the ash content increased in almost all samples except for the sample irradiated at 0.5 kGy, with a pronounced rise observed in the control samples (Fig. 1c).

### 3.6 Total moisture content

The moisture content of all samples showed an increase after 15 days. While no significant differences were observed on day one, but noticeable changes emerged on day 15 (Fig. 1d).

### 3.7 Total sugar content (%)

**Fig. 2:**
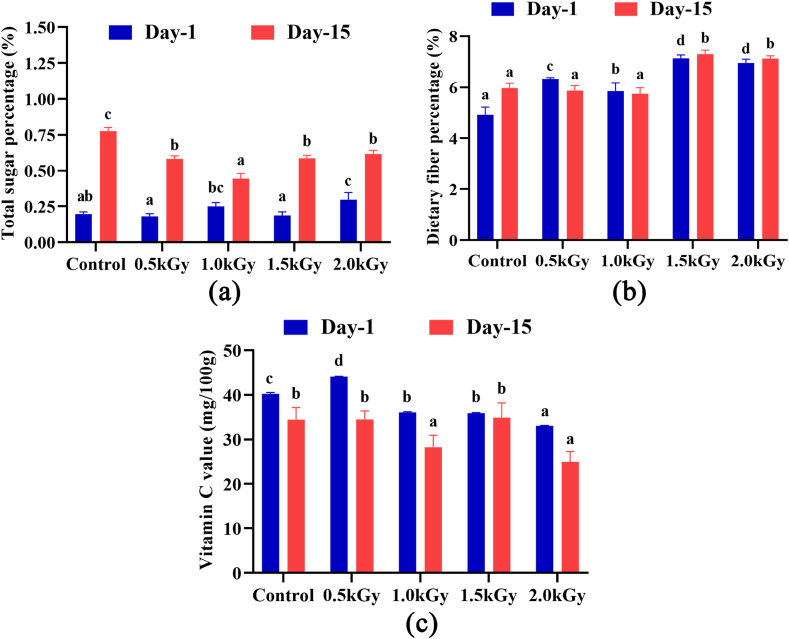
Total sugar, fiber, and Vit C content of the peel extract on day 1 and day 15. Different letters indicate significant differences between the mean ± SD of treatments (n = 3) at a P < 0.05 significance level.

Katimon mangoes are naturally sweet when ripe and fresh. The sugar content of the peel samples ranged from 0.1 to 0.3%, indicating their inherent sweetness. All samples exhibited an increase in sugar content after 15 days. Notably, the non-irradiated samples contained higher sugar levels compared to the irradiated ones. Significant variations were evident among the samples on both day one and day 15 (Fig. 2a).

### 3.8 Dietary fiber analysis

Fig. 2b illustrates the dietary fiber content in the Katimon peel samples. Following a 15-day of storage period, there were no significant differences in the dietary fiber levels between the control and irradiated peel samples.

### 3.9 Vitamin C amount analysis

The presence of ascorbic acid was found at trace levels in the 2 kGy-irradiated sample on day one amounting to 33.05 mg/100 g. The level of vitamin C in all samples had significantly decreased after 15 days compared to day one (Fig. 2c).

### 3.10 Estimation of titratable acidity

**Fig. 3:**
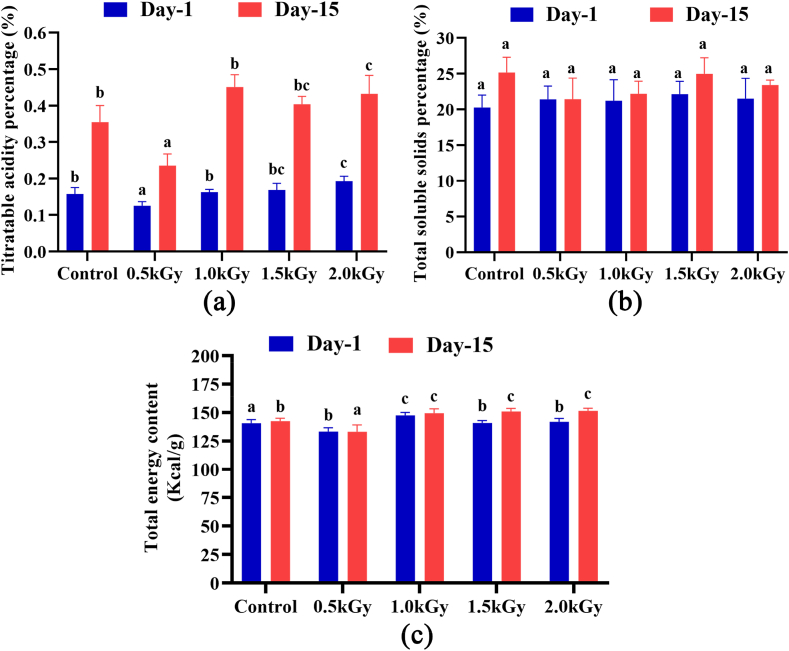
Total titratable acid, soluble solids, and total energy percentage of the peel extract on day 1 and day 15. Different letters indicate significant differences between the mean ± SD of treatments (n = 3) at a P < 0.05 significance level.

On day one, Katimon peel samples showed significant changes in titratable acidity (TA) content. After 15 days, titratable acidity content was increased in all the samples and changed significantly. Among the samples, the one subjected to 2 kGy radiation demonstrated the highest TA content (Fig. 3a).

### 3.11 Measurement of total soluble solids (TSS)

The sample irradiated with a 1.5 kGy dose rate exhibited the highest percentage (21.87 %) of total soluble solids (TSS). The remaining values were relatively consistent across all samples. Although no significant changes were observed among the samples on day 1 and day 15, an overall increase in TSS content was noted in all samples after 15 days of storage (Fig. 3b).

### 3.12 Estimation of energy

The sample exposed to a 1 kGy dose rate had a slightly higher energy content of 147.43 kcal/g. Fig. 3c depicts the fluctuating energy levels of peels subjected to varying doses after 15-day storage period. Some significant changes were found after 15 days of storage.

### 3.13 Antibacterial activity of *Mangifera indica* peel extracts

Antibacterial activity was assessed using different concentrations of peel extract. The control sample exhibited lower antibacterial activity. *Escherichia coli* and *Salmonella* sp. displayed intermediate resistance at doses of 100 and 150 μg/disc from peel extract. *Pseudomonas* sp. demonstrated notable susceptibility to irradiated samples, particularly at the 1.5 kGy radiation dose, where inhibition zones of 15.78 and 16.90 mm were recorded for 100 and 150 μg/disc doses (Table 3). Notably, *Escherichia coli* displayed resistance to this sample, while *Salmonella* sp. exhibited susceptibility at a dose rate of 150 μg/ml (Table 3).

**Table 3:** Inhibition zone (diameter in mm) with four doses of Mangifera indica peel extracts and kanamycin-30 μg (as control) against *Pseudomonas* sp., *Escherichia coli*, and *Salmonella* sp. Different letters used to indicate significant differences between the mean ± SD of replications (n = 3) at a significance level of P < 0.05.

Mango varieties	Sample Dose (μg per disc)	Mean Value of Inhibition Zone with Disc Diameter (mm)		
		*Pseudomonas* sp.	*Escherichia coli*	*Salmonella* sp.
**Katimon Control**	25	8 ± 0.06^a^	8 ± 0.28^a^	8 ± 0.2^a^
	50	8 ± 0.2^a^	9 ± 0.36^a^	8 ± 0.21^a^
	100	10.12 ± 0.5^b^	11.78 ± 0.65^b^	8.56 ± 0.3^a^
	150	10.26 ± 0.49^b^	10.89 ± 0.43^b^	10.05 ± 0.46^b^
	**Kanamycin 30 μg**	24.35 ± 0.34^c^	23.67 ± 0.88^c^	20.14 ± 0.6^c^
**Katimon 0.5 kGy**	25	10.14 ± 0.34^a^	8 ± 0.25^a^	8 ± 0.06^a^
	50	11.43 ± 0.41^b^	8 ± 0.1^a^	8 ± 0.025^a^
	100	15.57 ± 0.48^c^	9.10 ± 0.4^a^	9 ± 0.12^a^
	150	16.59 ± 0.63^c^	11.1 ± 0.14^b^	10.78 ± 0.59^b^
	**Kanamycin 30 μg**	22.15 ± 0.86^d^	22.23 ± 0.9^c^	21.34 ± 0.85^c^
**Katimon 1.0 kGy**	25	9 ± 0.17^a^	8 ± 0.15^a^	9 ± 0.02^a^
	50	9.45 ± 0.27^a^	8 ± 0.06^a^	9 ± 0.27^a^
	100	11.67 ± 0.58^b^	10.1 ± 0.26^b^	9.54 ± 0.46^a^
	150	15.11 ± 0.29^c^	10 ± 0.095^b^	11 ± 0.13^b^
	**Kanamycin 30 μg**	22.23 ± 0.43^d^	22.16 ± 0.46^c^	21.47 ± 0.46^c^
**Katimon 1.5 kGy**	25	10 ± 0.07^a^	8 ± 0.06^a^	9 ± 0.23^a^
	50	10 ± 0.37^a^	8 ± 0.21^a^	9.24 ± 0.22^a^
	100	15.78 ± 1.09^b^	9.89 ± 0.36^b^	10 ± 0.46^b^
	150	16.90 ± 0.35^b^	10.1 ± 0.13^b^	15.76 ± 1.10^c^
	**Kanamycin 30 μg**	22.33 ± 0.32^c^	22.25 ± 2.1^c^	21.56 ± 0.91^d^
**Katimon 2.0 kGy**	25	9.23 ± 0.46^a^	8 ± 0.05^a^	9 ± 0.02^a^
	50	11.2 ± 0.49^b^	8.11 ± 0.43^a^	9 ± 0.71^a^
	100	15.1 ± 0.75^c^	9 ± 0.03^b^	11.90 ± 0.34^b^
	150	15.89 ± 0.19^c^	10 ± 0.55^c^	15.78 ± 0.36^c^
	**Kanamycin 30 μg**	22.54 ± 1.48^d^	23.12 ± 0.58^d^	22.32 ± 0.3^d^

### 3.14 Antioxidant activity test

**Fig. 4:**
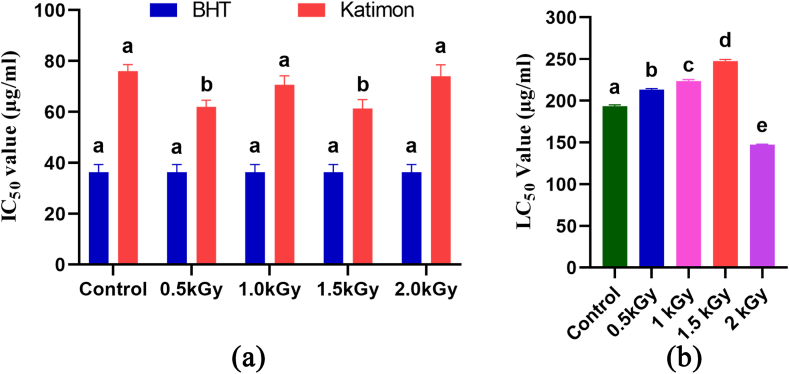
Effect of gamma radiation on antioxidant activity (a) and cytotoxic activity (b) of Katimon peel extract at different concentrations against *Artemia*. Different letters indicate significant differences between the mean ± SD of treatments (n = 3) at a P < 0.05 significance level.

This study examined the antioxidant impact (DPPH free radical scavenging activity) of BHT standard and peel extracts at three concentrations: 50 μg, 100 μg, and 150 μg/ml. The Katimon peel extract showed maximum antioxidant activity at 1.5 kGy radiation dose (Fig. 4a).

### 3.15 Cytotoxicity test

The cytotoxic impact of *Mangifera indica* peel extract was gauged using the Brine Shrimp (*Artemia salina*) lethality assay at various doses. Different peel extract types and doses yielded distinct LC_50_ values. Following a 24-h exposure, the concentration of 150 μg resulted in the highest mortality rate, while the concentration of 50 μg produced the lowest mortality rate (Fig. 4b).

### 3.16 Volatile compound analysis

Gas chromatography-mass spectrometry (GC-MS) analysis was employed to detect the volatile components in both the control (Fig. S1a) and the sample of Katimon mango variety that had undergone irradiation at 1.5 kGy (Fig. S1b). The irradiated sample exhibited elevated levels of various volatile compounds compared to the control group. Some of these compounds had antimicrobial properties, indicating that the microbial attack was significantly lower in the irradiated cultivars than in the control varieties.

### 3.17 Molecular docking study

**Fig. 5:**
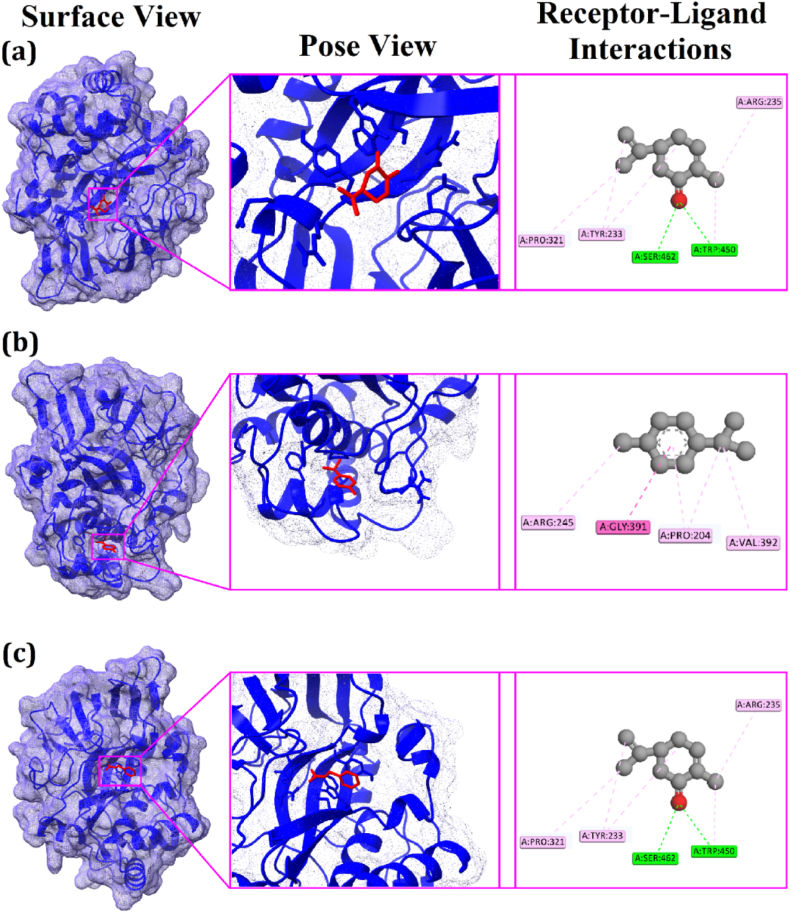
A docking simulation of TgpA protein with (−)-Carvone (a), *p*-Cymene (b), and Acetic acid, phenylmethyl ester (c), showing the surface view, pose view, and receptor-ligand interactions.

The virtual screening tool PyRx ensured a proper docking program that showed the binding affinity of the top three drug candidates (−)-Carvone, *p*-Cymene, Acetic acid, phenylmethyl ester with −6.2 kcal/mol, −6.1 kcal/mol, and −6.1 kcal/mol respectively (Table 4 and Table 5). In addition, BIOVIA Discovery Studio elicited the non-binding interaction between the top three candidates and the TgpA protein. In the first instance, (−)-Carvone was found to perform an interaction with TgpA in five sites where two conventional hydrogen bonds at TRP450 and SER462 position, two alkyl bonds at ARG235, PRO321 position, and one pi-alkyl bond at TYR233 position. The other drug candidate, *p*-Cymene expressed four interactions with TgpA where three alkyl bonds at ARG245, PRO204, and VAL392 residue position, and one amide-pi stacked bond at GLY391 position. In addition, the Acetic acid, phenylmethyl ester-TgpA complex stabilized by two conventional hydrogen bonds at ARG235 and SER462 position, one pi-pi stacked bond at the TYR233 position, one pi-pi T-shaped bond at the TRP450 position, and one alkyl bond at PRO321 position (Fig. 5a–c).

**Table 4:** Non-bond interactions between TgpA protein and the top three compounds.

Compound Name	Pubchem CID	Docking Score	Residues in Contact	Interaction Type	Distance in Å
(−)-Carvone	439,570	−6.2	TRP450	Conventional Hydrogen Bond	2.40796
			SER462	Conventional Hydrogen Bond	2.54326
			ARG235	Alkyl Bond	3.90929
			PRO321	Alkyl Bond	3.86921
			TYR233	Pi-Alkyl Bond	3.92452
*p*-Cymene	7463	−6.1	GLY391	Amide-Pi Stacked Bond	3.3168
			ARG245	Alkyl Bond	3.98014
			PRO204	Alkyl Bond	3.93272
			VAL392	Alkyl Bond	4.01469
Acetic acid, phenylmethyl ester	8785	−6.1	ARG235	Conventional Hydrogen Bond	2.2346
			SER462	Conventional Hydrogen Bond	1.83074
			TYR233	Pi-Pi Stacked Bond	3.37428
			TRP450	Pi-Pi T-shaped Bond	4.25037
			PRO321	Alkyl Bond	3.83144

**Table 5:** The top three candidates pharmacological profile.

Parameters	(−)-Carvone	*p*-Cymene	Acetic acid, phenylmethyl ester
Molecular weight	150.22	134.22	150.17
Num. H-bond acceptors	1	0	2
Num. H-bond donors	0	0	0
TPSA (S)	17.07 Å^2^	0.00 Å^2^	26.30 Å^2^
AMES toxicity	No	No	No
Human intestinal absorption	97.702 (% Absorbed)	93.544 (% Absorbed)	96.603 (% Absorbed)
Hepatotoxicity	No	No	No
P-glycoprotein substrate	No	No	No
Lipinski rule of five	Yes; 0 violation	Yes; 1 violation	Yes; 0 violation

### 3.18 ADMET analysis

Several ADMET properties of the top three ligands such as; molecular weight, number of hydrogen bond donors and acceptors, TPSA, different types of toxicity, intestinal adsorption, P-glycoprotein substrate, and Lipinski rule of five were evaluated (Table 5). The top three molecules, (−)-Carvone, *p*-Cymene, and Acetic acid as well as phenylmethyl ester, exhibited excellent results in the ADMET study. The molecular weight for (−)-Carvone, *p*-Cymene, and Acetic acid, phenylmethyl ester were 150.22 g/mol, 134.22 g/mol, and 150.17 g/mol respectively. The molecular weight of these molecules was perfect as compounds with higher MWs (MW > 500 g/mol) hinder the Lipinski rule of five [54,55]. Compounds should not contain more than five hydrogen bond donors and ten hydrogen bond acceptors to follow Lipinski's rule [55]. Number of hydrogen bond acceptors for (−)-Carvone was 1, for *p*-Cymene was 0 and for Acetic acid, Phenylmethyl ester was 2 and the number of hydrogen bond donors was 0 for these three lead molecules. To be considered a potential drug candidate, the topological polar surface area (TPSA) score must fall between 0 Å^2^ to 140 Å^2^ [[56], [57]]. Topological polar surface area (TPSA) was 17.07 Å^2^, 0.00 Å^2^, and 26.30 Å^2^ for (−)-Carvone, *p*-Cymene and Acetic acid, phenylmethyl ester respectively. Hepatotoxicity and AMES were not observed with these hit molecules, which suggests they could be effective drug candidates. Human intestinal absorption of (−)-Carvone was 97.702 %, *p*-Cymene was 93.544 %, and Acetic acid, Phenylmethyl ester was 96.603 %. Furthermore, these molecules were also recognized as non-substrates of P-glycoprotein. Besides, (−)-Carvone, *p*-Cymene, and Acetic acid, phenylmethyl ester all followed Lipinski's rule of five, although *p*-Cymene had 1 violation which is always acceptable for considering the drug-likeness property.

### 3.19 Molecular dynamics simulation

**Fig. 6:**
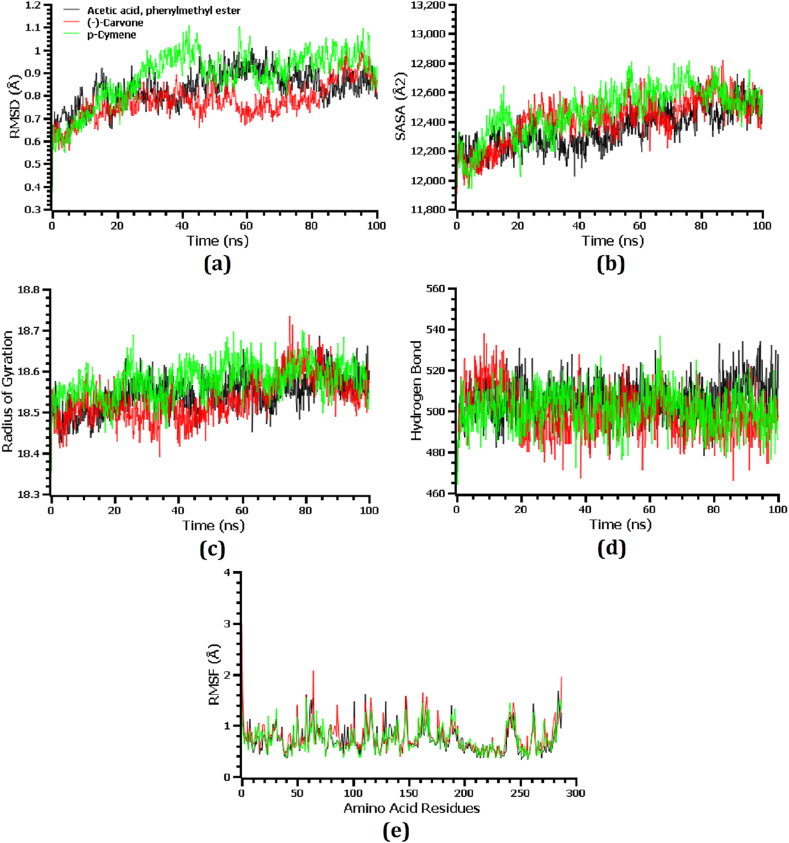
(a) Root mean square deviation, (b) solvent accessible surface area, (c) radius of gyration, (d) number of hydrogen bonds, and (e) root mean square fluctuation calculated from the molecular dynamics simulation of the top three compound-TgpA protein complex.

Molecular dynamics simulations were conducted to scrutinize the structural rigidity and validate docking scenarios for the top three protein-ligand complexes. By measuring the RMSD of C-alpha atoms, the stability of protein-ligand complexes was examined. Root Mean Square Deviation (RMSD) of complexes containing Acetic acid, Phenylmethyl ester, (−)-Carvone, and *p*-Cymene was initially increased due to this complex's instability, as shown in Fig. 6 (a). There was a higher rise in RMSD value on average in the *p*-Cymene-TgpA complex than in the other two complexes, whereas (−)-Carvone-TgpA complex had a lower average RMSD than the other two complexes. During the 45ns simulation time, the RMSd profile of the *p*-Cymene-TgpA complex dropped dramatically, stabilizing at 70 ns, and remaining stable throughout the remaining simulation time with negligible changes. Root Mean Square Deviation (RMSD) profiles of the (−)-Carvone-TgpA complex were lower between 40 and 80 ns than those of the other complexes, which might explain their greater stability. The RMSD value of the Acetic acid, Phenylmethyl ester-TgpA complex was stabilized at 70 ns of simulation time and remained stable for the rest of the simulation with minor fluctuation. However, all three complexes demonstrated RMSD of less than 2.5 Å over the simulation period, indicating they remained stable [52].

Solvent accessible surface area (SASA) values were determined for the three top complexes to determine how the surfaces of TgpA protein change due to interactions with ligand molecules. Increased SASA results in increased protein surface area, while decreases in SASA result in truncations of proteins [48]. A greater SASA value would suggest a larger surface area for the *p*-Cymene-TgpA complex, which is evidenced by its higher SASA on average than the other two complexes during the simulation period [Fig. 6 (b)]. Solvent accessible surface area (SASA) was lower on average for Acetic acid, Phenylmethyl ester-TgpA complex than the other two complexes throughout the simulation period. Acetic acid, the Phenylmethyl ester-TgpA complex, the (−)-Carvone-TgpA complex and the *p*-Cymene-TgpA complex reached a steady state after 80 ns, 70 ns, and 65 ns of the simulation period respectively, and persisted stable with only slight variations throughout the remaining simulation period.

The compactness or labile nature of protein complexes was determined by using Radius of gyration (Rg) values. Lower Rg values indicate a stiffer simulated protein complex, while greater Rg values indicate a more labile simulated protein complex [45]. Acetic acid, the Phenylmethyl ester-TgpA complex and (−)-Carvone-TgpA complex displayed an initial increase of Rg value followed by a decrease of the Rg value. In contrast to the other two complexes, the (−)-Carvone-TgpA complex exhibited relatively low Rg at around 20–65 ns of the simulation period, representing a more stable structure of this complex [Fig. 6 (c)]. Besides, the *p*-Cymene-TgpA complex had greater Rg values on average, indicating the labile nature of the protein than the other two complexes. Aside from that, all three complexes had relatively lower Rg values with very slight fluctuation that indicates the complex's stability throughout the simulation.

Hydrogen bonds are crucial to upholding the integrity and stability of proteins, so the hydrogen bonding of these docked complexes was examined [58]. A significant amount of hydrogen bonds was formed by the acetic acid, phenylmethyl ester-TgpA complex, (−)-Carvone-TgpA complex, and *p*-Cymene-TgpA complex throughout the simulation period, indicating a very strong bond between these three ligands and TgpA protein [Fig. 6 (d)]. RMSF values of these three ligands and TgpA complexes were examined to gain a better understanding of TgpA's suppleness across amino acid residues. Except for the first amino acid residue, all amino acid residues in the top three complexes had RMSF profiles below 2.5. Based on the lower RMSF values of the top three docked complexes, it is clear that the complexes have a lower level of flexibility, as lower RMSF values are associated with a higher level of stability [48] [Fig. 6 (e)].

## 4 Discussion

Gamma radiation emerges as an effective means to reduce microbial loads, as evident from the reduction in microbial presence observed in treated samples [13,59]. The use of nutrient agar allowed a comprehensive assessment of bacterial burdens. Samples exposed to radiation doses of 1.5 kGy and 2 kGy showcased diminished microbial presence. Consistent with prior findings, Iqtedar and his colleagues reported a decline in microbial load in Pakistani mango cultivars exposed to 1 kGy radiation [19]. Notably, irradiated mangoes exhibited prolonged ripening periods without detrimental effects, whereas control mangoes succumbed to rot within two weeks. In our study, the texture and color of mangoes exposed to 1.5 kGy radiation remained similar to the control. Correspondingly, Mahto and Das reported similar outcomes, asserting that irradiation impeded carotenoid formation, resulting in greener peel color and lighter pulp shade [60].

In biochemical assays, 2 kGy irradiated sample exhibited higher fat content than its counterparts on day 1. Over 15 days, the fat content of every sample increased but the control sample's fat content also significantly rose. In a research study, researchers found a higher percentage of fat in raw and ripe mango peels than in the current study [61]. In comparison to Ashoush and Gadallah's 2011 study, the current study found decreased fat content in samples [62]. After 15 days of storage, certain samples' protein content grew, while other samples' protein content declined. In Indian mango peels, Ajila et al. discovered a comparable range of protein concentrations [63].

Notably, the 1.5 kGy irradiated sample exhibited the highest ash percentage. Variations in ash content were apparent across samples, aligning with Ajila et al.'s observations [63]. It's worth noting that our study, unlike theirs, employed radiation to analyze ash content in mango peels. After 15 days, ash content increased in all samples, with control samples showing the most pronounced elevation. Analogous ash percentages were reported by Wongkaew et al. in their investigation of mango peels [64].

The quantity of moisture in the peel samples of our study is comparable to that of Indian Raspuri mango peels [63] and some Thai mango peels analyzed by Wongkaew et al. [64]. After 15 days, the moisture content of all samples increased, both in irradiated and non-radiated samples. A study found that both irradiated and non-irradiated bananas had the same moisture levels throughout storage [65].

Interestingly, sugar contents remained relatively constant between irradiated and control samples. All samples exhibited heightened sugar content after two weeks, with the control sample displaying the most substantial increase. Fluctuations in sugar content in climacteric fruits are attributed to maturation and consumption timing [66].

Dietary fiber content, averaging 5–7% across samples, demonstrated limited variation after 15 days in control peel samples. Contrasting with certain scholars who found no distinct fiber differences between gamma radiation-exposed and unexposed date varieties, our results showcase dietary fiber's resilience to radiation effects [67]. Ashoush et al. reported higher nutritional fiber in Indian mango peel acetone powder [62].

Among Katimon peel samples, control peels exhibited the highest ascorbic acid concentration. Iradiated samples displayed reduced Ascorbic acid levels, albeit radiation's influence on vitamin C content remained modest. Vitamin C content experienced a significant drop after 15 days, a phenomenon observed by others as well [68]. Ascorbic acid content was notably higher in mango peels, as per Tokas et al.'s study [69].

The total soluble solid content observed in both irradiated and non-irradiated Katimon peel samples ranged from 3.98 % to 4.89 %, which falls below the quantities reported by Tokas et al. in their investigation of Amrapali and Dasheri mango peel samples [69]. Titrable acidity values were notably higher in Katimon mango peel samples after a two-week storage period, albeit slightly lower than those found by Ajila et al. in their study [70]. Notably, elevated titrable acidity contributes to achieving an optimal flavor balance [69].

A study conducted by Lebaka and others, found a lower amount of carbohydrates in the mango peel [71]. Our findings reveal that protein, ash, and moisture levels all experienced elevation alongside carbohydrate content in several samples after 15 days. In terms of energy production, the Katimon cultivar irradiated with a 2 kGy dose rate yielded the highest energy levels in peel samples. Similarly, Ara et al.'s work with Bangladeshi mangoes echoes our results, lending support to the outcomes of this study [22]. Post 15 days of storage, the energy levels of distinct mango peel samples subjected to varying radiation doses underwent modifications.

Antibacterial potency evaluation involved assessing the aqueous extracts of Mangifera indica peel (Katimon) against three pathogenic bacteria strains: *Escherichia coli, Pseudomonas* sp., and *Salmonella* sp. Intriguingly, the Katimon peel sample irradiated with 1.5 kGy demonstrated maximum inhibition zones against *Pseudomonas* sp. Additionally, *Pseudomonas* sp. and *Salmonella* sp. exhibited susceptibility to the peel extract irradiated with 2.0 kGy. However, antibacterial activity against *Escherichia coli* was not evident. This diverges from Thambi and his teams' findings of mango peel extract's antibacterial activity against *Salmonella* and *E. coli* [72]. Furthermore, a separate study unveiled the antibacterial and antifungal potential of ethanolic peel extracts against pathogenic strains including *E. coli, Pseudomonas aerogenes*, and *Bacillus cereus* [73].

The antioxidant activity of Katimon control samples was evident, with the 1.5 kGy-irradiated sample displaying the highest antioxidant potential among irradiated samples. These findings mirror Ashoush and Gadallah's 2011 research, which affirmed mango peel's antioxidant activity and highlighted its positive impact on baked goods [74]. Similar conclusions were drawn in another study on macaroni production [75].

Evaluating cytotoxicity using the Brine Shrimp (*Artemia salina*) lethality assay indicated that the 1.5 kGy-irradiated Katimon sample demonstrated lower toxicity compared to other samples. In contrast, prior research has established mango peels' notable cytotoxicity levels, while mango flesh displayed comparatively lower cytotoxicity [76]. This disparity underscores the distinct nature of our findings.

Gas chromatography-mass spectrometry (GC-MS) study revealed a plethora of volatile compounds in Katimon mango variety, encompassing compounds like beta-myrcene, p-cresol, 3-*p*-Menthen-7-al, and other compounds. Some of these compounds have a wide range of insecticidal properties. Similar compounds were found by Laohakunjit and his team in their study [77]. Notably, while our study revealed modest fluctuations due to radiation, Fan & Sokorai reported substantial changes in volatile chemicals [78]. Similarly, Pino et al. identified compounds like menthol, limonene, and -pinene in Peruvian mangoes [79].

The in silico analysis was extended to assess antibacterial efficacy of peel extracts. It is imperative to examine the physicochemical attributes of novel drug leads to ascertain their potential drug likeness [80]. In this context, the molecular docking study scrutinized (−)-Carvone, *p*-Cymene, and Acetic acid, phenylmethyl as top compounds. The molecular weight of all three compounds adhered to Lipinski's rules, except for *p*-Cymene, which had a single violation. Moreover, the compounds exhibited a high absorption capacity and showed no toxicity. However, the study's limitations encompass restricted bacterial strain testing and the absence of consumer perception analysis due to sample size constraints, among other factors. To overcome these limitations and further advance this field, future research should focus on extended storage investigations, explore the utility of irradiated mangoes in value-added products, and encompass a wider spectrum of bacterial strains and fungi to gain a comprehensive understanding of radiation's effects on microorganisms.

## 5 Conclusion

The study reveals that gamma irradiation has significant effects on mango peel characteristics. It reduces microbial populations, maintains texture, stabilizes fat content, and impact sugar levels. Irradiated samples exhibit potential health benefits due to increased antioxidant activity. The molecular docking study identifies promising drug candidates with antimicrobial potential, supported by ADMET analysis for their suitability in drug development. Molecular dynamics simulations confirm stability of protein-ligand interactions. These findings provide a solid foundation for future research in the fields of food preservation, pharmaceuticals, and functional foods.

## Funding

The research was supported by the grants (696/5/52/Bio-11//R. U/2020–2021) provided by the 10.13039/501100016173University of Rajshahi, Bangladesh.

## Data availability statement

Data will be made available on request.

## CRediT authorship contribution statement

**Tabassum Jabin:** Formal analysis, Data curation. **Suvro Biswas:** Writing – original draft, Formal analysis, Data curation. **Shirmin Islam:** Methodology, Formal analysis, Data curation. **Swagotom Sarker:** Methodology, Investigation. **Mirola Afroze:** Methodology, Investigation. **Gobindo Kumar Paul:** Methodology, Formal analysis. **Mamudul Hasan Razu:** Resources, Methodology. **Md Monirruzzaman:** Resources, Investigation. **Mainul Huda:** Resources, Investigation. **Mashiur Rahman:** Methodology, Investigation. **Nayan Kumar Kundu:** Resources, Methodology. **Sabiha Kamal:** Methodology, Formal analysis. **Pranab Karmakar:** Software, Methodology. **Md Ariful Islam:** Methodology, Investigation, Formal analysis. **Md Abu Saleh:** Writing – review & editing, Supervision, Project administration, Conceptualization. **Mala Khan:** Writing – review & editing, Supervision, Resources. **Shahriar Zaman:** Writing – review & editing, Supervision, Project administration, Conceptualization.

## Declaration of competing interest

The authors declare that they have no known competing financial interests or personal relationships that could have appeared to influence the work reported in this paper.

## References

[1] Jahurul M.H.A. (2015). Mango (*Mangifera indica* L.) by-products and their valuable components: a review. Food Chem..

[2] Saleem Dar M., Oak P., Chidley H., Deshpande A., Giri A., Gupta V. (2015).

[3] Afifa K., Kamruzzaman M., Mahfuza I., Afzal H., Arzina H., Roksana H. (2014). A comparison with antioxidant and functional properties among five mango (Mangifera indica L.) varieties in Bangladesh. Int. Food Res. J..

[4] Rahman M., Consultancy R. (2021).

[5] Torres-León C., Rojas R., Serna-Cock L., Belmares-Cerda R., Aguilar C.N. (2017). Extraction of antioxidants from mango seed kernel: optimization assisted by microwave. Food Bioprod. Process..

[6] Serna-Cock L., García-Gonzales E., Torres-León C. (2016). Agro-industrial potential of the mango peel based on its nutritional and functional properties. Food Rev. Int..

[7] Kaur J. (2010). Preliminary investigation on the antibacterial activity of mango (*Mangifera indica* L : Anacardiaceae) seed kernel. Asian Pac. J. Tropical Med..

[8] Vega-Vega V. (2013). Antimicrobial and antioxidant properties of byproduct extracts of mango fruit. J. Appl. Bot. Food Qual..

[9] Gasu E.K., Appiah V., Gyamfi A.A., Nketsia-Tabiri J. (2012). Effects of irradiation and chemical preservatives on the microbiological quality of refrigerated fresh-cut mangoes. Eur. J. Food Res. Rev..

[10] Ashraf Chaudry M., Bibi N., Khan M., Khan M., Badshah A., Jamil Qureshi M. (2004). Irradiation treatment of minimally processed carrots for ensuring microbiological safety. Radiat. Phys. Chem..

[11] Santos G.H.F., Silva E.B., Silva B.L., Sena K.X.F.R., Lima C.S.A. (2011). Influence of gamma radiation on the antimicrobial activity of crude extracts of anacardium occidentale rich in tannins. Rev. Bras. Farmacogn..

[12] Zantar S. (2015). Effect of gamma irradiation on chemical composition, antimicrobial and antioxidant activities of Thymus vulgaris and Mentha pulegium essential oils. Radiat. Phys. Chem..

[13] Jabin T. (2023). Effect of gamma irradiation on chemical composition, antioxidant activity, antibacterial activity, shelf life, and cytotoxicity in the peels of two mango varieties grown in Bangladesh. Arab. J. Chem..

[14] Maldonado-Celis M.E. (2019). Chemical Composition of mango (Mangifera indica L.) fruit: nutritional and phytochemical compounds. Front. Plant Sci..

[15] Oliver-Simancas R., Díaz-Maroto M.C., Pérez-Coello M.S., Alañón M.E. (2020). Viability of pre-treatment drying methods on mango peel by-products to preserve flavouring active compounds for its revalorisation. J. Food Eng..

[16] Sarkar T., Bharadwaj K.K., Salauddin M., Pati S., Chakraborty R. (2022). Phytochemical Characterization, antioxidant, anti-inflammatory, anti-diabetic properties, molecular docking, pharmacokinetic profiling, and network pharmacology analysis of the major phytoconstituents of raw and differently dried Mangifera indica Himsaga. Appl. Biochem. Biotechnol..

[17] Rifaioglu A.S., Atas H., Martin M.J., Cetin-Atalay R., Atalay V., Doǧan T. (2019). Recent applications of deep learning and machine intelligence on in silico drug discovery: methods, tools and databases. Briefings Bioinf..

[18] Narsaiah K., Jha S.N., Jaiswal P., Singh A.K., Gupta M., Bhardwaj R. (2012). Scientia Horticulturae estimation of total bacteria on mango surface by using ATP bioluminescence. Sci. Hortic. (Amsterdam).

[19] Iqtedar M. (2016).

[20] Mahmud S., Shibly A.Z., Hossain M. (2015). The effects of CaC 2 and different calcium salt on mango fruits ripening in Bangladesh.

[21] International A. (1970).

[22] Ara R., Motalab M., Uddin M.N., Fakhruddin A.N.M., Saha B.K. (2014). Nutritional evaluation of different mango varieties available in Bangladesh. Int. Food Res. J..

[23] Janarthanam B., Sumathi E. (2010). Antimicrobial activity of Gymnema sylvestre leaf and callus extracts. J. Trop. Med. Plants.

[24] Choi C., Chae C. (2000). Distribution of porcine parvovirus in porcine circovirus 2-infected pigs with postweaning multisystemic wasting syndrome as shown by in-situ hybridization. J. Comp. Pathol..

[25] Meyer B.N., Ferrigni N.A., Putnam J.E., Jacobsen L.B., Nichols D.E., Mclaughlin J.L. (1982).

[26] Haocheng L., Kejing A., Su S., Yuanshan Y., X G., Jijun Wu Y.X. (2020). Aromatic characterization of Mangoes (*Mangifera indica* L .) using solid phase extraction coupled with. Foods.

[27] Kim S. (2019). PubChem substance and compound databases. Nucleic Acids Res..

[28] Hanwell M.D., Curtis D.E., Lonie D.C., Vandermeerschd T., Zurek E., Hutchison G.R. (2012). Avogadro: an advanced semantic chemical editor, visualization, and analysis platform. J. Cheminf..

[29] Studio D. (2015).

[30] Krieger G.V., Elmar, Spronk C. (2013).

[31] Krieger E., Vriend G. (2015). New ways to boost molecular dynamics simulations. J. Comput. Chem..

[32] Trott oleg, Olson Arthur J., Vina AutoDock (2010). Improving the speed and accuracy of docking with a new scoring function, efficient optimization, and multithreading. J. Comput. Chem..

[33] Wl D. (2002). The PyMOL molecular graphics system. CCP4 Newsl. Protein Crystallogr..

[34] Pettersen Ef Fau - Goddard T.D. (2004). UCSF Chimera--a visualization system for exploratory research and analysis. J. Comput. Chem..

[35] Cheng F. (2012). AdmetSAR: a comprehensive source and free tool for assessment of chemical ADMET properties. J. Chem. Inf. Model..

[36] Daina A., Michielin O., Zoete V. (2017). SwissADME: a free web tool to evaluate pharmacokinetics, drug-likeness and medicinal chemistry friendliness of small molecules. Sci. Rep..

[37] Pires D.E.V., Blundell T.L., Ascher D.B. (2015). pkCSM: predicting small-molecule pharmacokinetic and toxicity properties using graph-based signatures. J. Med. Chem..

[38] Land H., Humble M.S. (2018). YASARA: a tool to obtain structural guidance in biocatalytic investigations. Methods Mol. Biol..

[39] Wang J., Wolf R.M., Caldwell J.W., Kollman P.A., Case D.A. (2004). Development and testing of a general Amber force field. J. Comput. Chem..

[40] Mahmud S. (2021). Molecular docking and dynamics study to explore phytochemical ligand molecules against the main protease of SARS-CoV-2 from extensive phytochemical datasets. Expet Rev. Clin. Pharmacol..

[41] Harrach M.F., Drossel B. (2014). Structure and dynamics of TIP3P, TIP4P, and TIP5P water near smooth and atomistic walls of different hydroaffinity. J. Chem. Phys..

[42] Mahmud S. (2021). Prospective role of peptide-based antiviral therapy against the main protease of SARS-CoV-2. Front. Mol. Biosci..

[43] Biswas S. (2022). Molecular docking and dynamics studies to explore effective inhibitory peptides against the spike receptor binding domain of SARS-CoV-2. Front. Mol. Biosci..

[44] Mahmud S. (2021). Screening of potent phytochemical inhibitors against SARS-CoV-2 main protease: an integrative computational approach. Front. Bioinforma..

[45] Mahmud S. (2021). Plant-based phytochemical screening by targeting main protease of sars-cov-2 to design effective potent inhibitors. Biology.

[46] Essmann U., Perera L., Berkowitz M.L., Darden T., Lee H., Pedersen L.G. (1995). A smooth particle mesh Ewald method. J. Chem. Phys..

[47] Krieger E., Nielsen J.E., Spronk C.A.E.M., Vriend G. (2006). Fast empirical pKa prediction by Ewald summation. J. Mol. Graph. Model..

[48] Mahmud S. (2021). Efficacy of phytochemicals derived from avicennia officinalis for the management of covid-19: a combined in silico and biochemical study. Molecules.

[49] Kumar Paul G. (2022). Computational screening and biochemical analysis of Pistacia integerrima and Pandanus odorifer plants to find effective inhibitors against Receptor-Binding domain (RBD) of the spike protein of SARS-Cov-2. Arab. J. Chem..

[50] Mahmud S. (2021). Designing a multi - epitope vaccine candidate to combat MERS - CoV by employing an immunoinformatics approach. Sci. Rep..

[51] Mahmud S. (2022). Plant-derived compounds effectively inhibit the main protease of SARS-CoV-2: an in silico approach. PLoS One.

[52] Mahmud S. (2021). Antiviral peptides against the main protease of SARS-CoV-2: a molecular docking and dynamics study. Arab. J. Chem..

[53] Mahfuz A.M.U. (2022). In search of inhibitors of anti-cancer drug target Fibroblast Growth Factor Receptors: insights from virtual screening, molecular docking, and molecular dynamics study. Arab. J. Chem..

[54] Islam S. (2022). Biological efficacy of compounds from stingless honey and sting honey against two pathogenic bacteria: an in Vitro and in Silico study. Molecules.

[55] Lipinski C.A., Lombardo F., Dominy B.W., Feeney P.J. (2012). Experimental and computational approaches to estimate solubility and permeability in drug discovery and development settings. Adv. Drug Deliv. Rev..

[56] Mahmud S. (2022). Phytochemdb: a platform for virtual screening and computer-aided drug designing. Database.

[57] Jagannathan R. (2019). Characterization of drug-like chemical space for cytotoxic marine metabolites using multivariate methods. ACS Omega.

[58] Islam S., Mahmud L., Almalki W.H., Biswas S., Islam A. (2022).

[59] Khalaf H.A., El-Saadani R.M., El-Desouky A.I., Abdeldaiem M.H., Elmehy M.E. (2018). Antioxidant and antimicrobial activity of gamma-irradiated chicory (Cichorium intybus L.) leaves and roots. J. Food Meas. Char..

[60] Mahto R., Das M. (2013). Postharvest biology and technology effect of gamma irradiation on the physico-chemical and visual properties of mango (Mangifera indica L .), cv . ‘ Dushehri ’ and ‘ Fazli ’ stored at 20 ◦ C. Postharvest Biol. Technol..

[61] Marçal S., Pintado M. (2021). Trends in food science & technology mango peels as food ingredient/additive : nutritional value , processing , safety and applications. Trends Food Sci. Technol..

[62] Ashoush I.S., Gadallah M.G.E. (2011). Utilization of mango peels and seed kernels powders as sources of phytochemicals in biscuit. World J. Dairy Food Sci..

[63] Ajila C.M., Naidu K.A., Bhat S.G., Rao U.J.S.P. (2007). Food chemistry bioactive compounds and antioxidant potential of mango peel extract.

[64] Wongkaew M. (2021).

[65] Hassan P. (2007).

[66] Khan Q.U. (2018). Effect of gamma irradiation on nutrients and shelf life of peach stored at ambient temperature. Open Conf. Proc. J..

[67] ullah Mohammadzai I., Shah Z., Ihsanullah I., Khan H., Khan H., Rashid H. (2010). Effect of gamma irradiation, packaging and storage on the nutrients and shelf life of palm dates. J. Food Process. Preserv..

[68] Youssef B.M., Asker A.A., El-samahy S.K., Swailam H.M. (2002).

[69] Tokas J., Punia H., Baloda S., Rn S. (2020). Mango peel : a potential source of bioactive compounds and phytochemicals. Austin Food Sci.

[70] Ajila C.M., Aalami M., Leelavathi K., Rao U.J.S.P. (2010). Mango peel powder: a potential source of antioxidant and dietary fiber in macaroni preparations. Innov. Food Sci. Emerg. Technol..

[71] Lebaka V.R., Wee Y.J., Ye W., Korivi M. (2021). Nutritional composition and bioactive compounds in three different parts of mango fruit. Int. J. Environ. Res. Publ. Health.

[72] Thambi P.A., John S., Lydia E., Iyer P., Monica S.J. (2016). Antimicrobial efficacy of mango peel powder and formulation of recipes using mango peel powder (*Mangifera indica* L.). Int. J. Home Sci. IJHS.

[73] Mohamed Atta El-Desoukey R., Mohammed Aljor N., Daifallah Alaotibi A. (2020). The phytochemical and antimicrobial effect of mango (*Mangifera Indica* L.) peel extracts on some animal pathogens as eco-friendly. Acta Sci. Microbiol..

[74] Ashoush I.S., Gadallah M. (2011).

[75] Science I.F., Ajila C.M., Aalami M., Resources N., Krishnarau L. (2019).

[76] Mehjabin S., Akanda K.M., Mosaddik A., Parvez G.M.M. (2018).

[77] Laohakunjit N., Uthairatakij A., Kerdchoechuen O., Chatpaisarn A., Photchanachai S. (2005).

[78] Fan X., Sokorai K.J.B. (2002). Changes in volatile compounds of γ-irradiated fresh cilantro leaves during cold storage. J. Agric. Food Chem..

[79] Pino J.A., Mesa J., Muñoz Y., Martí M.P., Marbot R. (2005). Volatile components from mango (*Mangifera indica* L.) cultivars. J. Agric. Food Chem..

[80] Abdelfattah M.A.O. (2022). Antioxidant and anti-aging effects of Warburgia salutaris bark aqueous extract: evidences from in silico, in vitro and in vivo studies. J. Ethnopharmacol..

