# Supplementary material for: Effects of gamma-radiation on microbial, nutritional, and functional properties of Katimon mango peels: A combined biochemical and in silico studies
Source: Heliyon. 2023 Oct 31;9(11):e21556. doi: 10.1016/j.heliyon.2023.e21556 (PMC10665690; doi:10.1016/j.heliyon.2023.e21556)
Supplement: Multimedia component 1 [file mmc1.docx]

**Effects of gamma-radiation on microbial, nutritional, and functional properties of Katimon mango peels: A combined biochemical and in silico studies**

Tabassum Jabin^1,2^, Suvro Biswas^1^, Shirmin Islam^1^, Swagotom Sarker^2^, Mirola Afroze^2^, Gobindo Kumar Paul^1^, Mamudul Hasan Razu^2^, Md. Monirruzzaman^2^, Mainul Huda^2^, Mashiur Rahman^2^, Nayan Kumer Kundu^2^, Sabiha Kamal^2^, Pranab Karmakar^2^, Md. Ariful Islam^1^, Md. Abu Saleh^1,*^, Mala Khan^2,*^, Shahriar Zaman^1,*^

^1^Microbiology Laboratory, Department of Genetic Engineering and Biotechnology, University of Rajshahi, Bangladesh

^2^Bangladesh Reference Institute for Chemical Measurements (BRiCM), Dhaka, Bangladesh

| *Correspondence to: | Md. Abu Saleh ([saleh@ru.ac.bd](mailto:saleh@ru.ac.bd))  Professor, Department of Genetic Engineering and Biotechnology, University of Rajshahi, Bangladesh  Phone number: +8801716731747 |
| --- | --- |
|  | Mala Khan ([malakhan_07@yahoo.com](mailto:malakhan_07@yahoo.com)) |
|  | Shahriar Zaman ([szaman@ru.ac.bd](mailto:szaman@ru.ac.bd)) |


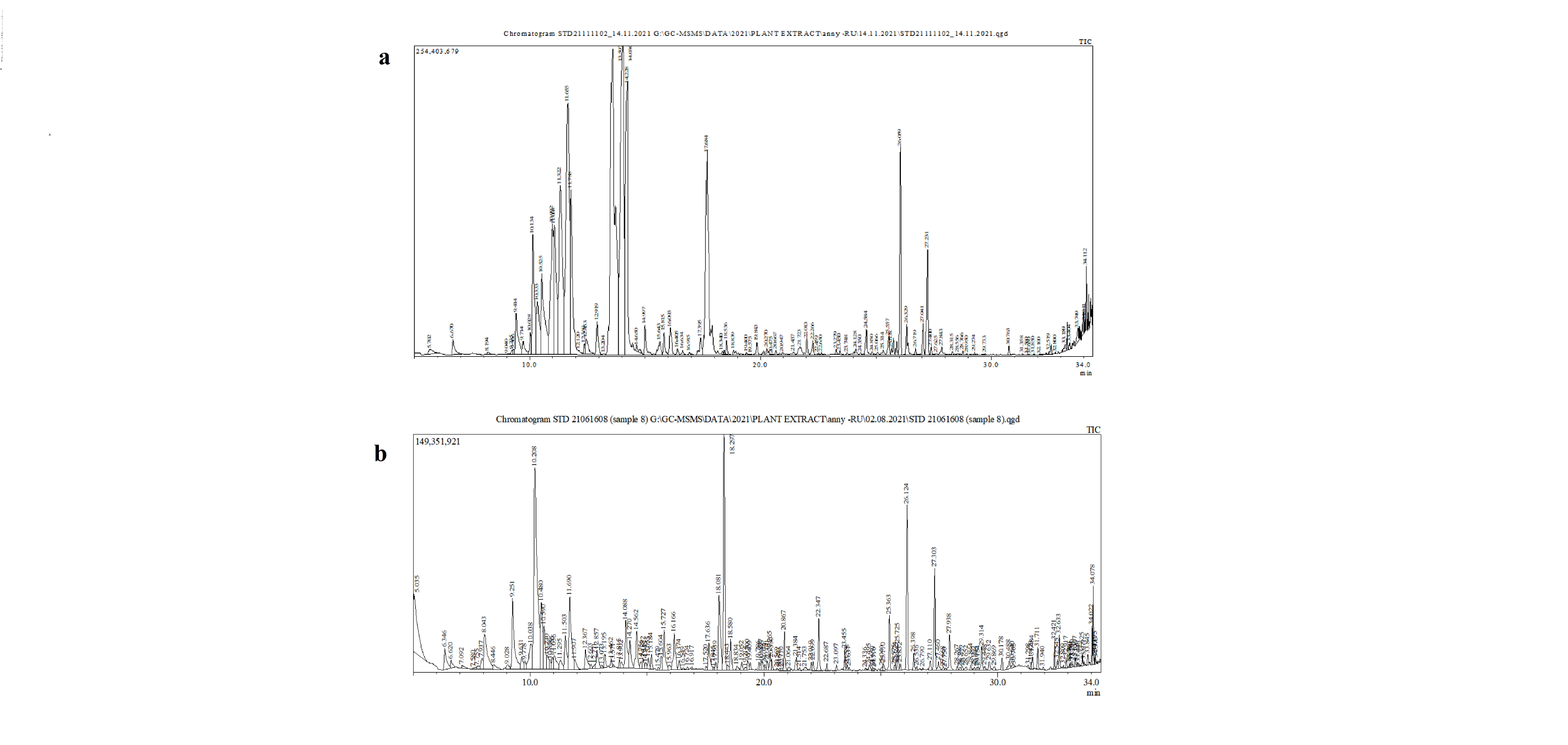


Fig S1: GC-MS chromatogram of Katimon control (a) and (b) 1.5 kGy radiated peel extract.

Table S1: Volatile compounds of treated and untreated *Mangifera indica* (Katimon) peel extracts

| Mango Varieties | Katimon Control | Katimon 1.5kGy |
| --- | --- | --- |
| Compound Name | Citronellol | Decanoic acid, octyl ester |
|  | beta.-Myrcene | Butyrolactone |
|  | (+)-4-Carene | Benzaldehyde |
|  | 2,4-Nonadiyne | (2,4,6-Trimethylcyclohexyl) methanol |
|  | 1,3,8-p-Menthatriene | Phytol |
|  | D-Limonene | Tetradecane |
|  | o-Cymene | Butanoic acid, butyl ester |
|  | 3-Carene | Hexanoic acid, ethyl ester |
|  | trans-Linalool oxide (furanoid) | D-Limonene |
|  | p-Cresol | p-Cymene |
|  | p-Mentha-1,5,8-triene | D-Limonene |
|  | 3-p-Menthen-7-al | Benzyl alcohol |
|  | 11-Methyldodecanol | 2-Furancarboxylic acid, ethyl ester |
|  | (2,4,6-Trimethylcyclohexyl) methanol | Butanoic acid, 3-methylbutyl ester |
|  | 4-Methylphenyl acetone | 2,4-Nonadiyne |
|  | Ascaridole epoxide | 4-Heptanone |
|  | 1-Chloro-2-methyl-2-phenylpropane | o-Cymene |
|  | 3-Methyl-2-(2-methyl-2-butenyl)-furan | 2,4-Hexadienoic acid, ethyl ester |
|  | Thymol | Butanoic acid, 3-hydroxy-, butyl ester |
|  | 2-Allyl-4-methylphenol | Tetrahydrofuran-2-acetic acid ethyl ester |
|  | 2-tert-Butyl-6-methylphenol, n-pentylether | Acetic acid, phenylmethyl ester |
|  | Caryophyllene | 3,7-Dimethyloct-6-en-1-yl decanoate |
|  | Humulene | Naphthalene |
|  | 2,4-Di-tert-butylphenol | Citronellol |
|  | 2-Bromotetradecane | (-)-Carvone |
|  | Decanoic acid, octyl ester | 11-Methyldodecanol |
|  | Dodecanoic acid, ethyl ester | Hexanoic acid, 3-hydroxy-, ethyl ester |
|  | Heptadecane | 3-Cyclohexene-1-carboxylic acid |
|  | 1-Heptatriacotanol | Glycerol 1,2-diacetate |
|  | Benzyl decyl maleate | Dodecanoic acid, ethyl ester |
|  | Dodecanoic acid, ethyl ester | Heptadecane |

Table S2: Docking results of the 1.5 kGy radiated peel extract.

| **Compound Name** | **Pubchem CID** | **Docking Score** |
| --- | --- | --- |
| (-)-Carvone | 439570 | -6.2 |
| (2,4,6-Trimethylcyclohexyl) methanol | 549904 | -5.8 |
| 11-Methyldodecanol | 33865 | -5.2 |
| 2,4-Hexadienoic acid, ethyl ester | 1550470 | -4.9 |
| 2,4-Nonadiyne | 569814 | -5 |
| 2-Furancarboxylic acid, ethyl ester | 11980 | -5.2 |
| 3,7-Dimethyloct-6-en-1-yl decanoate | 57353225 | -5.5 |
| 3-Cyclohexene-1-carboxylic acid | 20903 | -4.9 |
| 4-Heptanone | 31246 | -4.4 |
| Acetic acid, phenylmethyl ester | 8785 | -6.1 |
| Benzaldehyde | 240 | -5.2 |
| Benzyl alcohol | 244 | -5.1 |
| Butanoic acid, 3-hydroxy-, butyl ester | 103808 | -4.6 |
| Butanoic acid, 3-methylbutyl ester | 7795 | -5 |
| Butanoic acid, butyl ester | 7983 | -4.6 |
| Butyrolactone | 7302 | -4 |
| Citronellol | 8842 | -5.3 |
| Decanoic acid, octyl ester | 75321 | -5.2 |
| D-Limonene | 440917 | -5.7 |
| Dodecanoic acid, ethyl ester | 7800 | -5.3 |
| Glycerol 1,2-diacetate | 66021 | -5.4 |
| Heptadecane | 12398 | -5.1 |
| Hexanoic acid, 3-hydroxy-, ethyl ester | 61293 | -4.7 |
| Hexanoic acid, ethyl ester | 31265 | -4.6 |
| Naphthalene | 931 | -5.9 |
| o-Cymene | 10703 | -5.7 |
| p-Cymene | 7463 | -6.1 |
| Phytol | 5280435 | -5.5 |
| Tetradecane | 12389 | -5.3 |
| Tetrahydrofuran-2-acetic acid ethyl ester | 551345 | -5.1 |
